# Mucoadhesive Chitosan Delivery System with *Chelidonii Herba* Lyophilized Extract as a Promising Strategy for Vaginitis Treatment

**DOI:** 10.3390/jcm9041208

**Published:** 2020-04-22

**Authors:** Magdalena Paczkowska, Justyna Chanaj-Kaczmarek, Aleksandra Romaniuk-Drapała, Błażej Rubiś, Daria Szymanowska, Joanna Kobus-Cisowska, Emilia Szymańska, Katarzyna Winnicka, Judyta Cielecka-Piontek

**Affiliations:** 1 Department of Pharmacognosy, Faculty of Pharmacy, Poznan University of Medical Sciences, Swiecickiego 4, 60781 Poznan, Poland; mpaczkowska@ump.edu.pl (M.P.); justyna.chanaj-kaczmarek@ump.edu.pl (J.C.-K.); 2 Department of Clinical Chemistry and Molecular Diagnostics, Poznan University of Medical Sciences, Przybyszewskiego 49, 60355 Poznań, Poland; aromaniuk@ump.edu.pl (A.R.-D.); blazejr@ump.edu.pl (B.R.); 3 Department of Biotechnology and Food Microbiology, Poznan University of Life Sciences, Wojska Polskiego 48, 60627 Poznan, Poland; daria.szymanowska@up.poznan.pl; 4 Department of Gastronomy Science and Functional Foods, Poznan University of Life Sciences, Wojska Polskiego 28, 60637 Poznan, Poland; joanna.kobus-cisowska@up.poznan.pl; 5 Department of Pharmaceutical Technology, Faculty of Pharmacy, Medical University of Białystok, Mickiewicza 2c, 15222 Białystok, Poland; esz@umb.edu.pl (E.S.); kwin@umb.edu.pl (K.W.)

**Keywords:** *Chelidonium majus*, chelidonine and sanguinarine, chitosan, MTT test, microbiological activity, dissolution, permeability, ex vivo mucoadhesion

## Abstract

*Chelidonium majus* (also known as celandine) contains pharmacologically active compounds such as isoquinoline alkaloids (e.g., chelidonine, sanguinarine), flavonoids, saponins, carotenoids, and organic acids. Due to the presence of isoquinoline alkaloids, *Chelidonii herba* extracts are widely used as an antibacterial, antifungal, antiviral (including HSV-1 and HIV-1), and anti-inflammatory agent in the treatment of various diseases, while chitosan is a biocompatible and biodegradable carrier with valuable properties for mucoadhesive formulations preparation. Our work aimed to prepare mucoadhesive vaginal drug delivery systems composed of *Chelidonii herba* lyophilized extract and chitosan as an effective way to treat vaginitis. The pharmacological safety of usage of isoquinoline alkaloids, based on MTT test, were evaluated for the maximum doses 36.34 ± 0.29 µg/mL and 0.89 ± 1.16 µg/mL for chelidonine and sanguinarine, respectively. Dissolution rate profiles and permeability through artificial membranes for chelidonine and sanguinarine after their introduction into the chitosan system were studied. The low permeability for used save doses of isoquinoline alkaloids and results of microbiological studies allow confirmation that system *Chelidonii herba* lyophilized extract chitosan 80/500 1:1 (w/w) is a promising strategy for vaginal use. Ex vivo studies of mucoadhesive properties and evaluation of tableting features demonstrated that the formulation containing *Chelidonii herba* lyophilized extract (120.0 mg) with chitosan (80/500—100.0 mg) and polymer content (HPMC—100.0 mg, microcrystalline cellulose—50.0 mg, lactose monohydrate—30.0 mg and magnesium stearate—4.0 mg) is a vaginal dosage form with prolonging dissolution profile and high mucoadhesion properties (up to 4 h).

## 1. Introduction

Vaginitis is a prevalent disorder which affects millions of women. This is the key reason for women to visit their doctors. It is difficult to determine the true occurrence of vaginal infections, and doctors use the word vaginitis as a synonym for infections, believing that all vulvovaginal irritations or itching, especially when accompanied by irregular secretions, are caused by microorganisms [1,2]. Most specialists agree that up to 90% of vaginitis cases are due to bacterial vaginosis, vulvovaginal candidiasis, and trichomoniasis with the average incidence of bacterial vaginosis (BV) ranging from 9% to 50% [3,4,5,6]. In the US, bacterial vaginosis is actually the most severe cause of vaginitis and accounts for >30% of cases among child-bearing women [7]. Data suggest that approximately 75% of women will encounter a vaginal infection during their lifespan and that 40% to 45% will have more than one case [8]. Luckily, less than 5% of women suffer over four fungal infections per year. During their lifetime, 75% of all women are expected to develop at least one *Candida* vaginal infection, and up to 45% have two or more [9].

One of the essential plants, which is commonly used in traditional and folk medicine throughout the world, is *Chelidonium majus* (in short *C. majus*) [10], which contains isoquinoline alkaloids (e.g., chelidonine, chelerythrine, sanguinarine) [10,11]. These alkaloids have significant properties (antibacterial, antifungal, antiviral (including HSV-1 and HIV-1) and anti-inflammatory) for the treatment of vaginal infections [12]. Currently, *C. majus* is not recommended for use internally because it is hepatotoxic, but, according to European Medicine Agency (EMA) report, the use of its unique properties in topical preparations is still valuable and approved especially for the treatment of difficult viral infections [10,11,12,13]. Therefore alcoholic and oil extracts of *C. majus* are traditionally used externally to remove warts and corns (Belgium, Hungary, Spain), as well as to relieve psoriasis symptoms and to treat herpes around the mouth (Hungary) [10]. Hydroalcoholic extracts or squeezed milk juice from *C. majus* are used in the treatment of warts and condylomas, as well as in fungal and bacterial skin infections. Isolated *C majus* alkaloids—sanguinarine and chelerythrine—in the form of an ointment can be used in trichomoniasis of the vagina, cervix, vulva, and urethral opening [14]. However, there are still there no preparations that would be suitable for vaginal administration that would allow the valuable therapeutic properties of alkaloids to be used.

Formulations containing sanguinarine, chelerythrine, or chelidonine or their salts or extracts, including those mixed with suitable vehicles and/or excipients, are available for the treatment of common skin warts and verrucas, anal, vulvar warts, and psoriatic plaques [15]. For internal blemishes, the compounds according to the invention can be formulated as vaginal pessaries or suppositories or equivalent formulations for vaginal or anal treatment, including capsules that dissolve at internal body temperature. Considering that the deficit of vaginal preparations with a broad spectrum of activity exhibiting antibacterial, antifungal, and anti-cytomegalovirus activity seems to be justified in the development of preparations containing benzophenanthridine alkaloids for the treatment of vaginal infections with chronic inflammation [16].

Most of the products for vaginitis treatment are targeted at specific bacteria or fungi. *C. majus*-based medicine shows antibacterial, antifungal, and antiviral activity simultaneously. The advantages of such wide-range treatment are convenience, cost, and non-delayed accurate diagnosis and proper treatment. Hyaluronic acid is another preparation used for intimate infections. it regenerates, protects, and moisturizes, and additionally strengthens the walls of the vagina, moisturizes them, and, at the same time, makes them less susceptible to infection. However, hyaluronic acid does not show a wide spectrum of activity and is used as a means of preventing infection. In addition, *C. majus* is a raw material with a long history of use, which makes it safe with a known profile of side effects.

A vaginal route of drug administration serves as a potential site for both systematic and local action [17,18,19]. Unluckily, a significant limitation in the use of vaginal non-polysaccharides-based plant extracts is caused by a lack of adhesion to the vaginal mucosa [20,21]. Most of the dosage forms available currently on the market, such as liquid, semi-solid, and solid formulations, were conceived as immediate-release formulations [22]. Nevertheless, local vaginal tablets are relatively easy to insert and do not cause leakage. Moreover, tablets appear to be an appropriate therapeutic strategy aimed for the successful eradication of infectious reasons, the achievement of suitable drug levels at the target site, and simultaneously side effect minimization for the whole body [23]. A combination with a carrier of appropriate contribution to the vaginal mucosa and showing synergy of action allows them to prolong their activity, solving the non-adhesion limitations of herbal extract. Recently, a mucoadhesive polymer such as chitosan has been used for the preparation of bioadhesive vaginal tablet formulations [24,25,26]. Chitosan is a natural polycationic copolymer consisting of glucosamine and N-acetylglucosamine units, which is obtained by deacetylation of chitin derived mainly from the exoskeleton of crustaceans [27]. The suitability of chitosan for use in biomedical and pharmaceutical formulations is attributed to its inherent properties such as biodegradability, low toxicity, and excellent biocompatibility, which makes chitosan a valuable carrier [28,29]. Moreover, chitosan has antibacterial and antifungal activity, and due to its excellent mucoadhesive properties can be considered a suitable carrier to prepare buccal and vaginal dosage forms [25,30].

To the best of our knowledge, no reports have been presented on the preparation process of *Chelidonii herba* lyophilized extract with chitosan for vaginal use. Therefore our work aimed to develop mucoadhesive vaginal drug delivery systems composed of *Chelidonii herba* lyophilized extract with chitosan carrier.

## 2. Experimental Section

### 2.1. Chemicals and Instruments

Chelidonine and sanguinarine as phyproof^®^ Reference Substance were supplied by Sigma-Aldrich (Poznan, Poland). Ammonium hydroxide, potassium dihydrogen phosphate, methylene chloride, ethanol 96%, sulfuric acid 95%, and acetic acid 99.5% were purchased by Avantor Performance Materials Poland S.A. (Gliwice, Poland) HPLC-grade acetonitrile was obtained by Merck (Warsaw, Poland). Ammonium acetate was supplied by Chempur (Piekary Slaskie, Poland). Silicon dioxide (Aerosil^®^200), (hydroxypropyl)methylcellulose (HPMC) average Mn ~90,000, microcrystalline cellulose (Avicel^®^ PH-102), lactose monohydrate, magnesium stearate, and MTT solution were obtained from Sigma-Aldrich (Poznan, Poland). Chitosan 80/500 (degree of deacetylation: 77.6%–82.5%; viscosity: 351–750 mPas) and 80/1000 (degree of deacetylation: 77.6%–82.5%; viscosity: 751–1250 mPas) were supplied by Heppe Medical Chitosan GmbH (Halle, Germany). Prisma™ HT buffer, Acceptor Sink Buffer, GIT lipid solution were obtained by Pion Inc. (Billerica, USA). High-quality pure water and ultra-high-quality pure water were prepared by using a Direct-Q 3 UV Merck Millipore purification system (Merck, Warsaw, Poland).

### 2.2. Preparation and Analysis of Chelidonii herba Extract

#### 2.2.1. Plant Material

Plant raw material, herb of *C.majus (Chelidonii herba)*, was purchased from Herbapol Cracow (Cracow, Poland), Lot No: 010518, expiry date: 04.2021, and then ground into a powder.

Total alkaloids content in the plant raw material was determined according to The European Pharmacopoeia (*Chelidonii herba* monograph) [31]. Content of alkaloids was declared as not less than 0.6% of alkaloids sum, expressed as chelidonine.

#### 2.2.2. Extract Preparation and Freeze-Drying

Three hundred grams of dried herb of *C. majus* was extracted three times with an ethanol–water mixture (7:3 v/v) for 30 min at 95 °C on a water bath. The obtained extracts were collected and concentrated on a vacuum evaporator at a temperature below 35 °C to a volume of approx. 500 mL (BÜCHI Rotavapor R-210).

Then, the extract was frozen and lyophilized (CHRIST 1-4 LSC, Osterode am Harz, Germany). The temperature on the freeze dryer shelf was heated and ranged from +15 °C to +20 °C, the temperature inside the product was estimated as −4 °C, and the condensation temperature was set to −48 °C. The freeze-drying was conducted at reduced pressure (1.030 mbar) for 48 h.

Due to high hygroscopicity, the lyophilized extract was suspended in an aqueous solution of Aerosil^®^200 in a ratio of 5:1 (w/w) and again lyophilized. The obtained lyophilisate was sieved through a 0.49 mm sieve to get a powder. Next, in this powder, the content of active compounds (chelidonine and sanguinarine) was examined.

#### 2.2.3. Determination of Chelidonine and Sanguinarine Content in Chelidonii herba Lyophilized Extract

The concentrations of active compounds were determined by using the Ultra-High Performance Liquid Chromatography (UHPLC) method described previously by Gu et al. [32].

As equipment, LC system (Dionex Thermoline Fisher Scientific, Waltham, USA) with Chromeleon software version 7.0 was used. Separations were performed on an ACQUITY UPLC BEH C18 1.7 μm (2.1 × 100 mm) column. The detection of compounds was performed using a diode array detector at a wavelength maxima (λ_max_) at 280 nm. The following modifications in Gu’s method were done: the mobile phase was composed of ammonium acetate (10 mmol/L, adjusted to pH 3.0 with acetic acid) (A) acetonitrile (B) with a gradient elution: 0–0.8 min, 3% B; 0.8–1 min, 3%–12% B; 1–9.5 min, 12% B; 9.5–15 min, 12%–20% B; 15–20 min, 20%–30% B; 20–25 min, 30% B; 25 min, 3% B; 25–30 min, 3% B. The flow rate of the mobile phase was 0.4 mL/min, and the column temperature was maintained at 35 °C. The sample injection volume was 5.0 µL.

The presence of chelidonine and sanguinarine in the extract was confirmed by comparison of retention time and UV spectra of analyzed substances with their reference standards. The UHPLC-DAD method was validated according to the International Conference on Harmonization Guideline Q2 [33] in regards to selectivity, linearity, intra- and inter-day precision, and limits of detection (LOD) and quantitation (LOQ).

#### 2.2.4. MTT Test

Cytotoxicity of *Chelidonii herba* lyophilized extract, chelidonine, and sanguinarine in non-tumorigenic MCF10A human breast epithelial cell line was assessed using the MTT test. The stock solutions of all compounds were prepared in DMSO for *Chelidonii herba* extract at a concentration of 100 mg/mL, for chelidonine at a concentration of 30 mg/mL, and for sanguinarine at a concentration of 3 mg/mL. The assay was performed as previously described [34]. Briefly, cells were exposed to *Chelidonii herba* lyophilized extract, chelidonine, or sanguinarine in the range of concentrations 0 to 500 µg/mL, 0 to 150 µM (0 to 53.46 µg/mL), and 0 to 5 µM (0 to 1.84 µg/mL), respectively. Briefly, a total of 5 × 10^3^ MCF10A cells were seeded into each well of 96-well plates in a total medium volume of 100 μL per well. Cells were exposed to the studied compounds for 24 h. The solvent, DMSO in concentration 0.20%, was also applied as a control. Subsequently, 10 μL of MTT solution (5 mg/mL) was added to each well. The cells were incubated at 37 °C for 4 h followed by 100 μL of solubilization buffer (10% SDS in 0.01 M HCl) addition. After a 16 h incubation period, cell viability was quantified using a LabsystemsMultiscan RC spectrophotometer. Three separate experiments were performed, with eight repeats for each concentration. IC50 values were calculated using CalcuSyn (Biosoft, Cambridge, UK), and the standard deviation was calculated using Excel software (Microsoft, Redmond, WA, USA).

#### 2.2.5. Permeability Studies

Permeability of chelidonine and sanguinarine was investigated through the artificial biological membrane using a PAMPA GIT model simulating the gastrointestinal tract. The system consisted of a 96-well microfilter plate and a 96-well filter plate and was divided into two chambers: a donor at the bottom and an acceptor at the top, separated by a 120-μm-thick microfilter disc coated with a 20% (w/v) dodecane solution of a lecithin mixture (Pion, Inc.). The donor solution was adjusted to pH 4.5. The standards were dissolved in the donor solution. The plates were put together and incubated at 37 °C for 3 h in a humidity-saturated atmosphere. The concentrations of chelidonine and sanguinarine in the donor and acceptor compartments were determined by using the UHPLC-DAD method. The apparent permeability coefficients (*P*_app_) were calculated from the following Equation (1):

(1) $$P_{\mathrm{app}}=\frac{-\ln\left(1-\frac{C_{A}}{C_{\mathrm{equilibrium}}}\right)}{S\times \left(\frac{1}{V_{D}}+\frac{1}{V_{A}}\right)\times t}$$

where *V*_D_—donor volume, *V*_A_—acceptor volume, *C*_equilibrium_—equilibrium concentration $C_{\mathrm{equilibrium}}=\frac{C_{D}\times V_{D}+C_{A}\times V_{A}}{V_{D}+V_{A}}$, *C*_D_—donor concentration, *C*_A_—acceptor concentration, *S*—membrane area, and *t*—incubation time (in seconds).

To verify that *P*_app_ determined for permeability was statistically different, an ANOVA test was used. Compounds with *P*_app_ < 1 × 10^−6^ cm/s are classified as low-permeable and those with *P*_app_ > 1 × 10^−6^ cm/s as high-permeable compounds [35].

### 2.3. Preformulation Studies of Chitosan Delivery System with Chelidonii herba Lyophilized Extract

#### 2.3.1. System Preparation

The standardized *Chelidonii herba* lyophilized extract was mixed in an agate mortar for 1 h with chitosan 80/500 and 80/1000 in weight ratio 1:1 (w/w) and 1:5 (w/w) to obtain a uniform yellow-brown powder. The obtained powder was stored at room temperature in a desiccator with limited air access.

#### 2.3.2. Dissolution Studies

The dissolution studies of chelidonine or sanguinarine from the chitosan matrix were conducted by using the Agilent 708-DS dissolution apparatus. A standard paddle method was used at 37 ± 0.5 °C with a stirring speed of 50 rpm. Phosphate buffer (pH 4.5) was used as an acceptor medium in a volume of 150 mL to assure sink conditions [36]. The liquid samples were collected at specified time intervals with the replacement of an equal volume of temperature-equilibrated media and filtered through a 0.45 μm membrane filter. The time points (0, 2, 5, 10, 15, 30, 45, 60, 90, 120, 150, 180 min) were the same for all formulations (up to 180 min). The concentrations of active compounds in acceptor solutions were determined by using the UHPLC-DAD method.

Release profiles were compared by using the model proposed by Moore and Flanner, which is based on two-factor values, *f*_1_ and *f*_2_ [37]. The difference factor (*f*_1_) measures the percentage error between two curves over all time points, and *f*_2_ is a logarithmic transformation of the sum-squared error of differences between the test *T_j_* and reference *R_j_* system over all time points according to the formulas below (Equations (2) and (3)):

(2) $$f_{1}=\frac{\sum_{j=1}^{n}\left|R_{j}-T_{j}\right|}{\sum_{j=1}^{n}R_{j}}\times 100$$

(3) $$f_{2}=50\times \log\left({\left(1+\left(\frac{1}{n}\right)\overset{n}{\underset{j=1}{\sum }}{\left|R_{j}-T_{j}\right|}^{2}\right)}^{-\frac{1}{2}}\times 100\right)$$

where *n* is the sampling number and *R_j_* and *T_j_* are the percentages dissolved of the reference and test products, respectively, at each time point *j*. Dissolution profiles are similar when the *f*_1_ value is close to 0 and *f*_2_ is close to 100 (FDA guidelines suggest that two profiles are identical if *f_2_* is between 50 and 100).

#### 2.3.3. Microbiological Activity

All test samples (lyophilized extract, chitosan systems as well as pure chitosans) were dissolved in 1.0 mL of dimethyl sulfoxide (DMSO) and mixed until fully dissolved. From so-obtained stock solutions at a concentration of 100 mg/mL, a series of dilutions in the concentration range 0.5–15 mg/mL in Antibiotic Broth medium (Merck) was prepared. For each l.0 mL of dilution, 0.1 mL of 18-hour-old liquid culture of standard strains, diluted 1:10,000 in the same Antibiotic Broth medium, was added (the number of added cells was approximately 103 in 0.1 mL). The samples were incubated at 37 °C for 24 h. After this time, all dilutions were inoculated on solid Antibiotic Agar. After a further 24 h, the lowest concentration of sample dilutions, which completely inhibited the reference strain growth, was marked as Minimal Bactericidal Concentration (MBC) [38].

### 2.4. Formulation Studies

Binary systems of mucoadhesive vaginal tablets were prepared containing *Chelidonii herba* lyophilized extract with chitosan 80/500 and following excipients: HPMC, microcrystalline cellulose, lactose monohydrate, and magnesium stearate in weight ratio 1:1 (w/w). Binary systems in a weight ratio from designed formulation were also made (Table 1).

These systems have been subjected to stability tests at room temperature at controlled air humidity (RH = 50%). At the appropriate time points (3, 6, and 12 months), concentrations of active compounds were measured by the UHPLC-DAD method.

#### 2.4.1. Tableting Process

Tablets containing 100.0 mg of *Chelidonii herba* lyophilized extract and selected excipients (Table 1) were prepared by direct compression using a laboratory scale, single punch tableting machine, NP-RD10A Tablet Press (Natoli, Saint Charles, MN, USA). Compaction properties of tablets (size 7 × 15 mm with concave oval profile) were assessed using several pressures in the range of 27 to 165 MPa.

#### 2.4.2. Tablet Characterization

Tablet characterization studies were conducted according to methods described in Ph. Eur. 9th [39]. Weight of freshly produced tablets was recorded immediately after compaction. Weight, thickness, and diameter were measured on 20 randomly selected tablets using a balance and manual vernier caliper. The hardness of the tablets was measured using the PTB-M manual tablet hardness testing instrument (Natoli). All measurements were followed by the calculation of mean values and standard deviations (SD).

Tensile strength (*σ*) represents the resistance of the tablets to fracturing. Tensile strength values were calculated from the Breaking Force (*F*) values (N), where *D* is the length of short-axis (mm), *t* is the overall thickness (mm), and *W* is wall height of the tablet (mm), using the following Equation (4) [40]:

(4) $$\sigma =\frac{2}{3}\left(\frac{10F}{\pi D^{2}\left(2.84\frac{t}{D}-0.126\frac{t}{W}+3.15\frac{W}{D}+0.01\right)}\right)$$

Solid fraction (*SF*), also known as relative density, was calculated by using the following equation, where *W_t_* is tablet weight (mg), *v* is the volume of a tablet, and *ρ_true_* is the true density of the powder (g/cm^3^). The true density (*ρ_true_*) of powders were measured using gas pycnometer AccyPyc II 1340 (Micrometrics) (Equation (5)):

(5) $$SF=\frac{W_{t}}{\rho_{true}v}$$

The tablet porosity (*ε*) was calculated from *SF* using the following Equation (6):

(6) ε=1−SF

#### 2.4.3. In Vitro Release Studies

The dissolution studies were conducted according to the procedure described in Section 2.3.2. The time points (0, 15, 30 and 45 min, and 1, 1.5, 2, 2.5, 3, 3.5, 4, 5, 6, 7, 8, 24 h) were the same for all tablets (up to 24 h).

The obtained active compound release profiles were fitted to the following mathematical models: zero-order equation: F=k×t; first-order equation: lnF=k×t; Higuchi equation: $F=kt^{1/2}$; Korsmeyer–Peppas equation: $F=kt^{n}$, where *F*—the fraction of release drug, *k*—the constant connected with release, and *t*—the time [41,42].

#### 2.4.4. Determination of the Ex Vivo Mucoadhesive Properties

Mucoadhesive properties of prepared tablets were studied in regard to the evaluation of maximum detachment force, work of adhesion, and residence time. Measurements were conducted using the TA.XT Plus texture analyzer (Stable Microsystems, Godalming, UK) equipped with a 5 kg load cell and measuring system G/muc (20 mm diameter). Porcine vaginal mucosa obtained from pigs weighing about 200 kg (Bost, Turosn Koscielna, Poland) was used. The studies did not require the approval of a local animal research ethics committee. The tissue obtained immediately after killing the animals was washed with fluid simulating vaginal secretion SVF (simulated vaginal fluid, pH 4.2), divided into fragments, and stored at −20 ± 2 °C until measurements were made.

Prior to tests, porcine vaginal mucosa was connected with cyanoacrylate adhesive to heating stainless steel plate and thermostated (37.0 ± 0.5 °C) for 5 min. The tablet was attached with a cyanoacrylate adhesive to the upper probe with a diameter of 20 mm and moistened with 100 μL phosphate buffer pH 4.5. The analyzer operating parameters, namely pre- and post-test speed 2 mm/s, contact force 0.3 N, and contact time 60 s, were selected during preliminary studies.

Using the Texture Exponent 32 computer program, the maximum force required to detach the tablet from the porcine vaginal mucosa *F*_max_ (mN) was recorded, and the work of mucoadhesion *W*_ad_ (μJ) was calculated from the area under the force versus distance curve. Cellulose acetate paper was used as a negative control.

The residence time of designed tablets to the porcine vaginal mucosa was determined using a modified apparatus for the disintegration time test described by Nakamura et al. [43]. Tissue fragments were attached to the inner surface of the beaker with a cyanoacrylate adhesive (length approx. 4–5 cm, width approx. 2–3 cm). The vessel filled with 500 mL phosphate buffer pH 4.5 was thermostated at 37 ± 1 °C for the period of time conducting research. The tablets were attached to the porcine mucosa by applying a force for 20 s. Next, a plexiglass cylinder (diameter 6 cm, weight 280 g) was placed in the apparatus, which, when started, moved cyclically up and down, ensuring complete immersion of the mucosa with the attached drug dosage form. The time needed to completely detach (or disintegrate) the drug from from vaginal mucosa was measured.

## 3. Results and Discussion

Due to the antimicrobial activity, it is worth considering plant raw material from *Chelidonii herba* as valuable in the treatment of infectious etiology diseases [9]. On the other hand, one cannot forget about systemic toxicity, which has been recently reported for this plant material [10]. Therefore under pharmacopoeial requirements, the total alkaloid content should be controlled for the safe use of *Chelidonii herba*. Bearing in mind the potential of pharmacological use of *C. majus*, but also the limitation of systemic use, the authors proposed a new approach to the therapeutic use of this plant material as mucoadhesive vaginal tablets. Vaginal infections are characterized by infectious etiology, which corresponds to the spectrum of microbial activity of *Chelidonii herba* [11]. By using topical application, local action is ensured, but at the same time it is possible to limit the penetration of isoquinoline alkaloids into the general bloodstream and as a consequence, to reduce their toxic effect.

This work aimed to prepare safe and effective mucoadhesive vaginal tablets containing release-modifying chitosan with *Chelidonii herba* lyophilized extract.

As the first stage of the experimental work, freeze-dried hydroalcoholic *Chelidonii herba* extract was prepared. The plant material used for these studies met the pharmacopoeial requirements. The total alkaloid content of *Chelidonii herba* was 0.97% ± 0.03%. According to Ph. Eur. 9th Edition, the herb of *C. majus* should have content of the sum of alkaloids calculated as chelidonine not less than 0.6% [32]. Due to the high hygroscopicity of the lyophilized extract, it was decided to mix it with Aerosil^®^200 in a weight ratio of 5:1 (w/w) and re-lyophilized.

**Figure 1:**
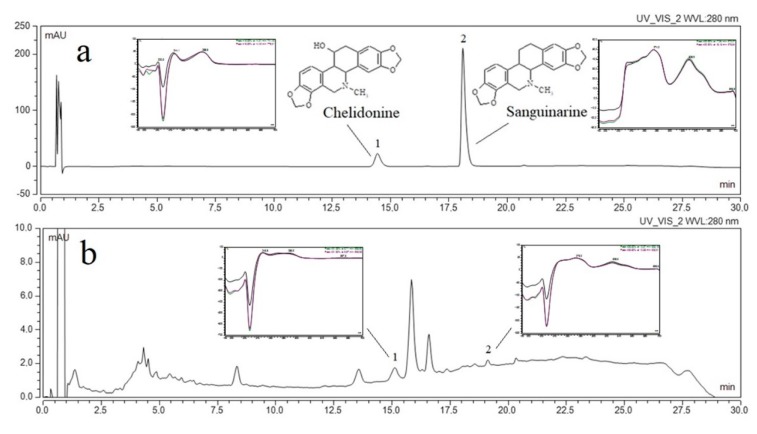
The UHPLC chromatogram of analytical standards (chelidonine (1) and sanguinarine (2)) (**a**) *Chelidonii herba* lyophilized extract (**b**).

The identification and the determination of active compounds (chelidonine and sanguinarine) contained in the plant material and *Chelidonii herba* lyophilized extract, as well as all concentration changes of chelidonine and sanguinarine during dissolution and permeability studies, was carried out using an ultra-high performance liquid chromatography supported by a photodiode array detector. The UHPLC-DAD method was developed according to the method described by Gu et al., which was modified in regards to the gradient elution and then validated according to ICH Q2 guidelines [33]. Validation parameters are presented in Supplementary materials (Table S1). Chelidonine and sanguinarine were dissolved in two different solvents: methanol (as extraction solvent) and a phosphate buffer with pH ~4.5 (as dissolution medium). The retention times of the peaks of chelidonine and sanguinarine were compared with the retention times of the reference substances (Figure 1) as well as their UV spectra. A linear relationship between the peak areas and the concentrations of chelidonine and sanguinarine dissolved in both media was obtained. The range of linearity for both alkaloids was the same, and the sensitivity of determinations was similar. The developed method may be the reference method for determining the concentration of both alkaloids in plant material and pharmaceutical dosage forms containing those alkaloids.

Using the linearity equation of reference substances dissolved in phosphate buffer, it was possible to determine the content of active compounds in the lyophilized extract. Chelidonine had a higher content (7.84 μg per 1 mg of the lyophilized extract), while the content of sanguinarine was 0.19 μg per 1 mg of lyophilized extract.

**Figure 2:**
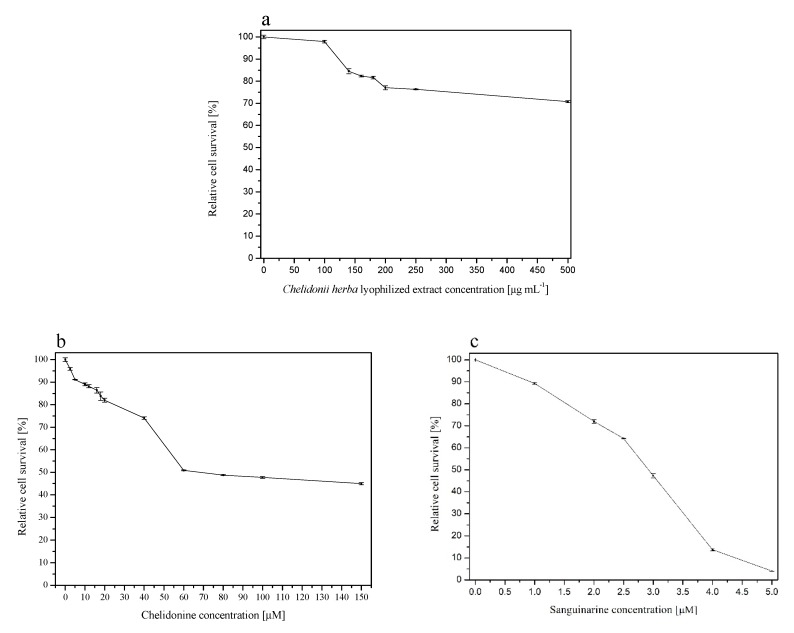
Cytotoxic effect of *Chelidonii herba* lyophilized extract (**a**), chelidonine (**b**), and sanguinarine (**c**) on MCF10A cells.

As mentioned, due to the potential hepatotoxic effect of the extract, it was essential to conduct screening tests to select a dose of alkaloids delivered form *Chelidonii herba* in pharmaceutical dosage forms. For this purpose, cytotoxicity tests and MTT test were carried out. Cytotoxicity assay revealed that *Chelidoni herba* extract significantly inhibited the survival of non-tumorigenic human epithelial cells MCF10A. An almost 20% cytotoxicity was observed when a concentration of 140, 160 or 180 µg/mL was applied (Figure 2a). Further concentrations of the studied compound provoked a higher cytotoxic effect, and it reached the value of almost 30% at the highest concentration, i.e., 500 µg/mL. Interestingly, when cells were treated with chelidonine alone, the cytotoxic effect was also significant, and it showed a dose-dependent effect in the whole range of the concentration, showing 5% survival decrease at the concentration of 2.5 µM and 55% decrease when the highest concentration (i.e., 150 µM) was applied (Figure 2b). However, sanguinarine appeared to be the most toxic and inhibited cell survival significantly in the range of 1 to 5 µM starting from 10% at the lowest and reaching a 95% decrease at the highest concentration of 5 µM (Figure 2c). The IC_50_ values calculated from the survival curves were as follows: 541.98 ± 3.30 µg/mL (*Chelidonii herba* lyophilized extract), 102.82 ± 0.83 µM or 36.34 ± 0.29 µg/mL (chelidonine), and 2.43 ± 3.15 µM or 0.89 ± 1.16 µg/mL (sanguinarine). Interestingly, the extract revealed lower toxicity compared to individual active compounds. Noteworthy, the content of chelidonine was 40-fold higher than the amount of sanguinarine per 1 mg of lyophilized extract (7.84 vs. 0.19 μg per 1 mg of lyophilized extract, respectively, as mentioned above). Surprisingly, sanguinarine exhibited 40-fold higher cytotoxicity based on IC_50_ value. Considering these observations, it is difficult to assess which of the isoquinoline alkaloids (chelidonine or sanguinarine) play a major role in the activity of the extract, but it may also be the matter of some interplay. Cytotoxicity test showed that all the studied compounds affected the survival of the MCF10A cells in vitro (Figure 2). The cytotoxic potential of *Chelidonii herba* derived compounds is not very well established. Scientific reports are showing some discrepant data indicating an impact of the compound on the proliferation potential of cells in vitro that is minor [44] or high enough to postulate some anticancer potential that is based on the decreased survival due to DNA damage effect [45]. Interestingly, sanguinarine is shown to reveal a significant antiproliferative potential through its action on the Na^+^-K^+^-ATPase transmembrane protein [46]. It is also suggested that the cytotoxic effect of *Chelidonii herba*-derived compounds may be due to autophagy induction resulting from alterations in cell membrane composition, activation of intracellular caspases, disruption of the mitochondrial membrane potential, and reactive oxygen species (ROS) generation [47]. Generally, these compounds are considered particularly cytotoxic against human cancer cells [45].

The vaginal route of drug administration serves a potential site for not only a local but also systematic absorption of a variety of therapeutic agents, especially for female-related conditions [18]. Therefore our research also involved assay of penetration of chelidonine and sanguinarine through biological membranes that stimulate the vaginal walls. Using the GIT PAMPA model, it was possible to study the permeability of chelidonine and sanguinarine by passive diffusion. As a result of a 3 h incubation, it was possible to determine the concentrations of reference substances and to calculate the apparent permeability value *P*_app_ for chelidonine (0.23 × 10^−6^ cm/s) and sanguinarine (0.16 × 10^−6^ cm/s). For both used doses of alkaloids, *P*_app_ values were less than 1 × 10^−6^ cm/s, which indicates a low permeability of the tested compounds through biological membranes [36]. The permeability of sanguinarine was about 1.5 times weaker than that of chelidonine. Based on the permeability values determined for the reference substances, the mass of lyophilized extract, necessary to obtain permeability of chelidonine and sanguinarine to the same extent, was calculated. The weight of the lyophilized extract would be 19.13 mg to provide *P*_app_ chelidonine equal to 0.23 × 10^−6^ cm/s and 794.27 mg to contain *P*_app_ sanguinarine equal to 0.16 × 10^−6^ cm/s. Our results were in agreement with ones obtained by Kosina et al. and Psotova et al. [48,49], where it was confirmed that chelidonine and sanguinarine are characterized by low absorption through biological membranes of the gastrointestinal tract. The obtained values indicate that the topical application of the tested lyophilized extract should exert local effect after topical use with low risk to exert a systemic influence. We can say that in the case of topical application of *Chelidonii herba* alkaloids, characterized by a low absorbing process, the cytotoxic effect may not show up at all.

For the preformulation studies, chitosan was selected as a carrier for the preparation of systems with the potential vaginal application due to its biodegradability and low toxicity, as well as its antibacterial and antifungal activity [29,30]. Chitosan binary systems were obtained by mixing of a *Chelidonii herba* lyophilized extract with chitosan 80/500 and 80/1000 in weight ratio 1:1 and 1:5 (w/w) to get a uniform powder.

**Figure 3:**
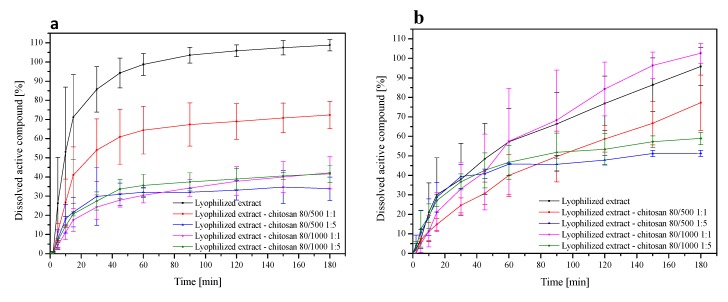
Dissolution profiles of the chelidonine (**a**) and the sanguinarine (**b**) from *Chelidonii herba* lyophilized extract at its chitosan delivery systems at pH ~4.5.

To examine the influence of chitosan on release profiles of chelidonine and sanguinarine from the *Chelidonii herba* lyophilized extract, dissolution studies were conducted. The dissolution studies in phosphate buffer at pH 4.5 simulating vaginal fluids were determined (Figure 3a,b). In the case of chelidonine release, after 90 min, 103.54% ± 4.10% of chelidonine was released from the lyophilized extract, while only 67.35% ± 11.28%, 32.04% ± 2.11%, 34.28% ± 4.31%, and 37.44% ± 4.73% for lyophilized extract-chitosan 80/500 1:1 (w/w), lyophilized extract-chitosan 80/500 1:5 (w/w), lyophilized extract-chitosan 80/1000 1:1 (w/w), and lyophilized extract-chitosan 80/1000 1:5 (w/w), respectively. Based on the obtained release profiles, it can be stated that over the entire duration of the test (180 min), 95.81% ± 9.70% sanguinarine was released from the lyophilized extract, while from chitosan systems, 77.24% ± 14.23%, 51.25% ± 1.60%, 102.60% ± 5.16%, and 58.92% ± 3.22%, from the following systems: lyophilized extract-chitosan 80/500 1:1 (w/w), lyophilized extract-chitosan 80/500 1:5 (w/w), lyophilized extract-chitosan 80/1000 1:1 (w/w), and lyophilized extract-chitosan 80/1000 1:1 (w/w), respectively.

The introduction of chelidonine into chitosan systems reduced and slowed down its release. Chelidonine released from the lyophilized extract was 80% within 30 min, whereas from chitosan systems, this release lasted 180 min and over. Similar correlations were observed in the case of sanguinarine release, where from the lyophilized extract, it was dissolved in over 90% within 180 min, and from chitosan systems, only 70% or less. Dissolution profiles for chelidonine and sanguinarine had different shapes (*f_1_* values above 15 and *f*_2_ less than 50). In the case of chelidonine, we observed slow release, which may be associated with its chemical structure. The hydroxyl group of chelidonine can interact with the amino groups of chitosan and bind with van der Waals interactions, which can result in a reduced degree of dissolution of chelidonine in dissolution media. Moreover, it was found that as the percentage of chitosan in the system increases, the dissolution rate of the reference substances decreases. The reason may be hydration of the outer layer of the system, which results in the formation of a gel layer on its surface [50]. This reduces the penetration of water into the core of the system and, as a result, may hinder the transport of reference substances, leading to their slow dissolution. The gradual dissolution of the active elements in this study may, therefore, be due to less water penetration into the core of the system [50,51].

Considering the beneficial pharmaceutical properties of chelidonine and sanguinarine systems with chitosan carriers, the potential of bactericidal and fungicidal activity for them was also evaluated. The most frequent causes of vaginitis are infections due to bacterial vaginosis (including *Gardnerella vaginalis* and *Mycoplasma hominis*), *Candida albicans* (yeast) infections, and *Trichomonas vaginalis* infections. A microbiological analysis to determine the minimal bactericidal concentration of lyophilized extract (control) and its binary systems with chitosan was determined for nine bacterial species, including clinical isolates (Table 2). Microbiological tests confirmed the activity of the lyophilized extract against positive bacteria, negative bacteria, and fungi. The registered bactericidal activity profile of the *Chelidonii herba* lyophilized extract is characterized by the spectrum of activity, which indicates that the lyophilization process does not lose the biological activity of the raw material [52].

The combination of the *Chelidonii herba* lyophilized extract and chitosan gives the possibility of a prolonged release of chelidonine and sanguinarine into the vaginal fluid, which is the subject for microbiological tests. The prepared chitosan systems with *Chelidonii herba* lyophilized extract were characterized by Minimal Bactericidal Concentration (MBC) values, which gave information about the microbial activity. The MBC value of *Chelidonii herba* lyophilized extract was considered as the reference one. It should be noted that the *Chelidonii herba* extract alone shows the highest activity against *S. pyogenes* (8 mg/mL) and *S. epidermidis* (8 mg/mL), while the lowest against *C. albicans* (125 mg/mL). For chitosan, microbiological activity is negligible. The creation of the chitosan delivery system with *Chelidonii herba* lyophilized extract did not lower the MBC value in most of the studied cases, and it even increased this indicator. Particularly promising were the results of microbiological activity of combinations of chitosan with *Chelidonii herba* lyophilized extract against *Pseudomonas aeruginosa* (16 mg/L for the lyophilized extract vs. 4 mg/L for lyophilized extract + chitosan 80/1000 1:1 (w/w)), infections of which are a significant clinical problem [53]. It is estimated that, currently, most of the available antibiotics have no effect against *Pseudomonas* spp. [54]. The very low MBC value of chitosan systems (8 mg/L) compared to the higher MBC values of the *Chelidonii herba* lyophilized extract (125 mg/L) in the case of *Candida albicans* seems to be promising strategy for fungal infections, especially since they are around 20% of all infections [55].

In the last stage of the work, optimization of obtaining a mucoadhesive, vaginal, pharmaceutical form containing the *Chelidonii herba* lyophilized extract with chitosan 80/500 1:1 (*w/w*) was carried out in order to obtain appropriate mucoadhesive properties to the vaginal mucosa. The chitosan delivery system 80/500 1:1 (w/w) was selected based on the most favorable release profile and microbial activity profile. Mucoadhesive formulations F1–F3 were prepared with a constant percentage of lyophilized extract (25%) and chitosan (25%) and changing the content of HPMC (0%–25%) (Table 1).

**Figure 4:**
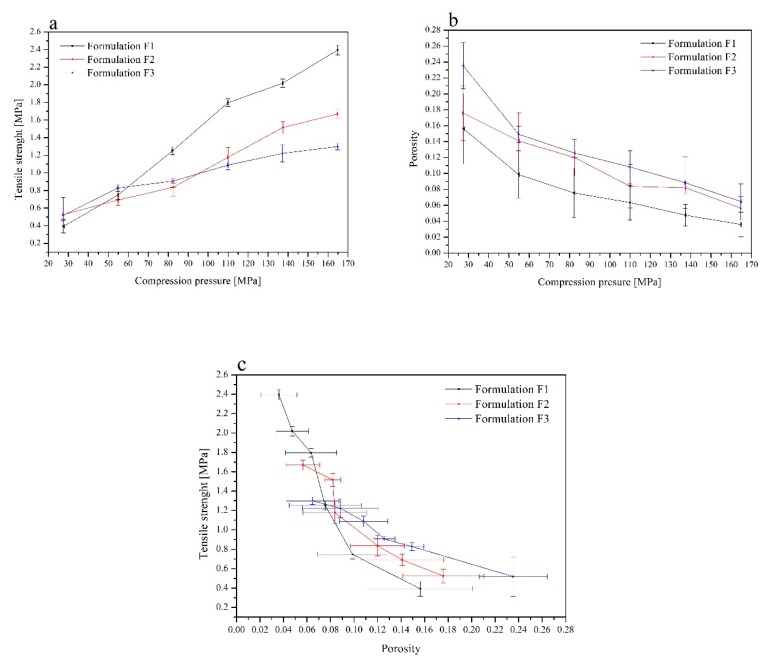
Tabletability (**a**), compressibility (**b**), and compactibility (**c**) profiles of the formulations F1–F3.

Tablet tensile strength, solid fraction, and porosity at a range of compression pressure are the most important parameters describing the material’s compaction properties. Tabletability is represented by a plot of compression pressure (MPa) versus the tensile strength (MPa), thus measuring the impact of increasing the compression force on the resulting tablet’s tensile strength. The relationship between tablet hardness and the compression force is almost linear over a broad range. From the mechanical strength evaluation, the formulation F1 provided the strongest compact (Figure 4a). Compressibility profile, plotting compaction pressure (MPa) versus porosity, assesses how readily the material undergoes a change in volume when compressed. For the formulation F1, the highest compaction pressure resulted in close to zero porosity. At a specific compaction pressure, the formulation F3 yields the highest porosity, and the formulation F2 provides very similar solid fraction results (Figure 4b). Compactibility profile is the tablet’s tensile strength as a function of the solid fraction (or porosity). Good compactibility describes a material capable of achieving desired tablet hardness at low compaction pressure. With this in mind, careful selection of optimum compaction pressures to be employed for tableting studies was imperative. When formulations were compacted at 110 MPa, a tensile strength of 1.8 MPa for the formulation F1, 1.2 MPa for F2, and 1.1 MPa for F3 was achieved. Statistical analysis confirmed the significant greater tensile strength of the formulation F1 over F2 and F3. This increased tensile strength of the formulation F1 tablets was accompanied by low porosity, ranging from 0.04 to 0.16, compared with the formulation of F2 and F3 tablets of 0.06 to 0.24 in the same pressure range. It is also important to remember that any unnecessary increase in compaction pressure can induce physical changes upon the compacted material. The presence of HPMC within tablets (formulation F2 and F3 versus F1) had a significant impact on the porosity of the compacts (Figure 4c). In the previous studies on tableting properties of chitosan carried out by Aucamp et al., it could be seen that chitosan could not be compressed into tablets on an eccentric tablet press [56]. Poor compressibility of chitosan, resulting in tablets with low crushing strength and relative high friability, was due to the high porosity of chitosan. Aucamp concluded that even if combining chitosan with fillers such as Avicel PH 200^®^ or Prosolv^®^ SMC 90 (co-processed microcrystalline cellulose and colloidal silicon dioxide) the tablet strength was still weak [57]. The combination of chitosan with the filler (Avicel PH 200^®^ or Prosolv^®^ SMC 90) in the ratio 7:3 (w/w) gave the best results. The conclusion was that the filler improved the flowability of the powder blend, resulting in better die filling and an increase in the tablet strength [56]. It only confirmed findings from these studies, where the best tableting properties were achieved for the formulation F3, where the highest amount of HPMC and other excipients was added.

**Figure 5:**
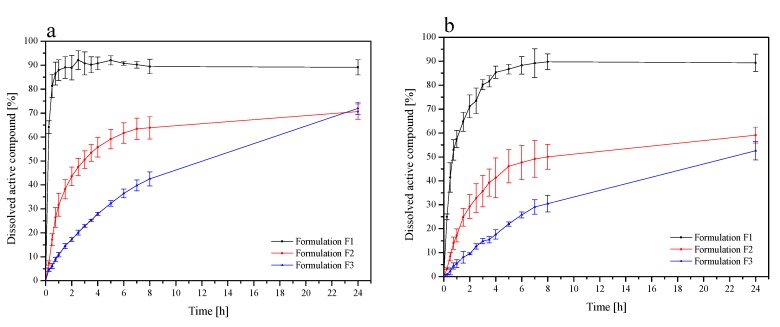
Dissolution profiles of chelidonine (**a**) and sanguinarine (**b**) from the chitosan tablets with *Chelidonii herba* lyophilized extract (F1–F3) at pH ~4.5.

The release kinetics of chelidonine and sanguinarine from chitosan tablets with *Chelidonii herba* lyophilized extract were determined (Figure 5a,b). In F1 formulations, where the interaction between chelidonine and sanguinarine with chitosan, due to the presence of basic groups, was the strongest release of alkaloids into the vaginal fluid. Increasing the HPMC content in F1 and F2 formulations reduced the release of alkaloids, increasing their activity at the site of adhesion. Chelidonine interacted less well with chitosan than sanguinarine, resulting in more dynamic release.

To investigate what mechanism is responsible for the prolonged release, dissolution data obtained for the formulation F2–F3 were fitted to the following release models of zero-order and first-order equations, Higuchi model (applied for matrix systems), and Korsmeyer-Peppas model (employed for swellable matrices) (Table 3).

As the most probable, two models of chelidonine and sanguinarine release Higuchi and Korsmeyer–Peppas were shown. The release exponent *n* was found to be below 0.5 in the formulations F2 and F3, indicating that the drug release followed the Fickian process—the term refers to a gradient-dependent release of alkaloids from tablets. Based on the regression correlation coefficient, it could be seen that both alkaloids released from tablets F2 and F3 were controlled by diffusion as it was best fitted to the Higuchi model (Table 3) [58]. Diffusion seems to be one of the essential processes to release active compounds from vaginal formulations [17].

**Figure 6:**
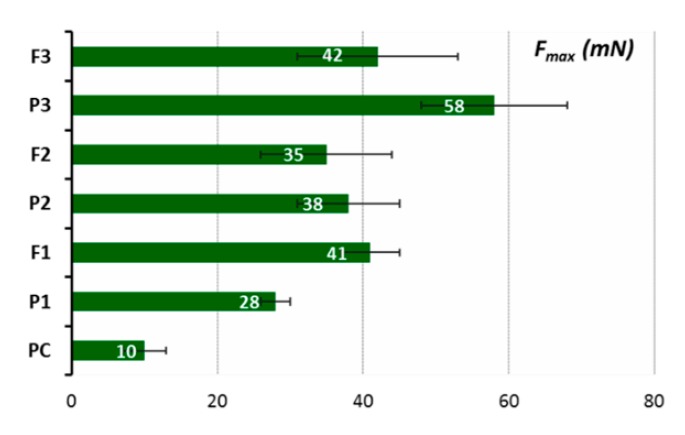
The maximum force of detachment (*F*_max_, mN) of placebo (P1–P3) and chitosan tablets with *Chelidonii herba* lyophilized extract (F1–F3) compared to cellulose acetate paper control (PC) (*n* = 4; mean ± SD).

**Figure 7:**
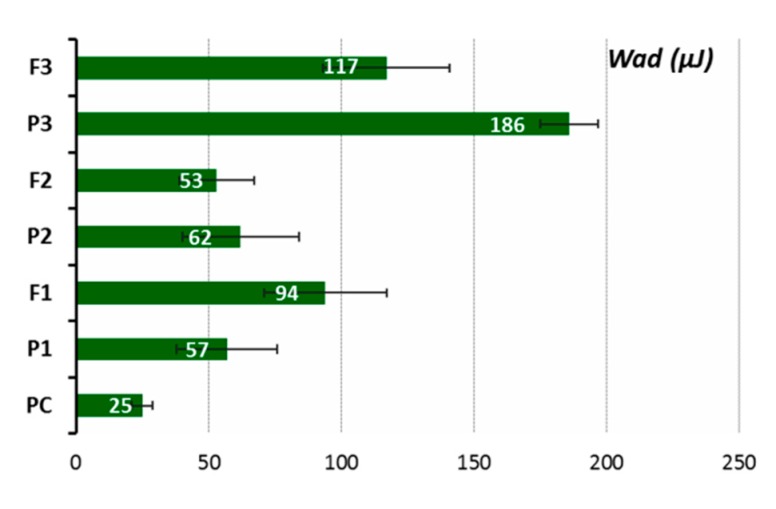
Mucoadhesion (*W*_ad_) placebo (P1–P3) and chitosan tablets with *Chelidonii herba* lyophilized extract (F1–F3) with porcine vaginal mucosa compared to cellulose acetate paper control (PC) (*n* = 4; mean ± SD).

Ex vivo mucoadhesive measurements of the vaginal tablet’s ability to adhere to mucosal tissue were performed applying two independent techniques. The influence of tablet composition, including chitosan and HPMC contents, on the force of detachment (*F*_max_) and work required to overcome the formulation–porcine-vaginal-mucosa interactions (*W*_ad_) is displayed in Figure 6 and Figure 7, whereas their residence time to animal tissue determined by using a self-constructed apparatus is shown in Table 4.

It was observed that all tablets adhered immediately to the mucosal surface, and all of them displayed a higher ability to interact with the porcine vaginal mucosa as compared to control, i.e., cellulose paper. Among alkaloids-loaded formulations, tablets F3 containing HMPC and chitosan in a 1:1 ratio (F3) exhibited the highest *F*_max_ and *W*_ad_ values. In general, the strength of the mucoadhesive bond grew by increasing the concentration of HPMC (F2 vs. F3) in formulations. Notably, the formulation F1 with chitosan possessed comparable or even higher values of *F*_max_ and *W*_ad_ as compared to the formulation F2 with chitosan/HPMC. The residence time studies confirmed that the formulation F3 displays greater mucoadhesiveness as it exhibited a profoundly higher contact time (up to 4 h) than the other formulations. Although all examined tablets adhered immediately to the mucosal tissue, the residence time of formulations F1 and F2 were limited as a result of their rapid disintegration in acceptor medium. The presence of herb extract was found to decrease the mucoadhesive capacity of polymers slightly but solely in the formulation F3 with chitosan/HPMC in ratio 1:1 (w/w).

Ex vivo mucoadhesive measurements showed differences in mucoadhesive behavior among tested tablets. The formulation F3 with chitosan/HPMC ratio 1:1 (w/w) displayed the highest ability to adhere to mucosal tissue (Figure 6 and Figure 7). During the initial wetting of tablets F2 and F3 with phosphate buffer, a viscous hydrophilic film appeared on the surface, the presence of which seems to favor the formation of interactions between the polymer chains and the mucous membrane. Additional cohesion forces appeared as a consequence of wetting dosage form with acceptor medium, which may have also supported the adhesion of tablets to animal tissue. The addition of lactose (about 25% and 20% of the tablet mass in F1 and F2 formations, respectively) probably could have been a factor limiting the interaction between chitosan and HPMC and biological material [59]. The results from additional residence time studies confirmed the best ability of formulation F3 to interact with the porcine vaginal mucosa (Table 4). Finally, the formulation F3 displayed adhesion time of 4 h, which appears advantageous for application reasons. According to literature data, the drug form should not stay in the vaginal cavity for more than 6 h due to the occurrence of discomfort and the risk of disturbance in vaginal secretion [60,61,62], so the tablets F3 meet these criteria. Comparable observations were obtained from the previous studies, where the chitosan/HPMC ratio played an essential role in mucoadhesive properties of the pharmaceutical formulation of chitosan tablets with clotrimazole [59]. The formulation F3 (chitosan/HPMC ratio 1:1) would be pressed into the tissue surface instead of interacting with only the mucin layer; consequently, this could result in a higher detachment force. The active compound release profiles, mucoadhesion force, and mucoadhesion residence times are the parameters used to select the best formulation, and the patent’s comfort is a factor that cannot be overlooked when seeking to improve adherence to the treatment.

## 4. Conclusions

The vaginal mucoadhesive tablets, containing systems of *Chelidonii herba* lyophilized extract with chitosan with an application algorithm with high compliance (e.g., two times a day), is a safe and effective approach to the treatment of vaginal infections of bacterial and fungal etiology. The combination of alkaloids with the chitosan carrier ensures appropriate synergy of action as well as mechanical properties of the prepared pharmaceutical dosage forms. To sum up, the developed mucoadhesive formulations are good candidates for an effective treatment of vaginal infection.

## Figures and Tables

**Table 1:** Composition chitosan tablet formulations with *Chelidonii herba* lyophilized extract.

	Placebo 1 (P1)	Formulation 1 (F1)	Placebo 2 (P2)	Formulation 2 (F2)	Placebo 3 (P3)	Formulation 3 (F3)
	Content (mg) of Compounds in One Tablet					
*Chelidonii herba* lyophilized extract + Aerosil 5:1	-	120.0	-	120.0	-	120.0
Chitosan 80/500	100.0	100.0	100.0	100.0	100.0	100.0
HPMC	-	-	50.0	50.0	100.0	100.0
Microcrystalline cellulose (Avicel 102)	50.0	50.0	50.0	50.0	50.0	50.0
Lactose monohydrate	130.0	130.0	80.0	80.0	30.0	30.0
Magnesium stearate	4.0	4.0	4.0	4.0	4.0	4.0
Sum	284.0	404.0	284.0	404.0	284.0	404.0

**Table 2:** MBC values of *Chelidonii herba* lyophilized extract and its chitosan delivery systems.

Microorganism	*Chelidonii herba* Lyophilized Extract	Lyophilized Extract + Chitosan 80/500 1:1	Lyophilized Extract + Chitosan 80/500 1:5	Lyophilized Extract + Chitosan 80/1000 1:1	Lyophilized Extract + Chitosan 80/1000 1:5	Chitosan 80/500	Chitosan 80/1000
	MBC (mg/mL)						
*S. aureus*	8	16	125	64	125	125	250
*S. epidermidis*	8	16	125	64	125	125	250
*E. faecalis*	32	125	125	32	64	125	250
*S. pyogenes*	8	32	32	16	125	125	250
*E. coli*	32	64	64	32	125	62	125
*P. aeruginosa*	16	16	125	4 ↓	62	125	125
*C. albicans*	125	64 ↓	125	8 ↓	125	250	250

↓ - decrease in MIC value.

**Table 3:** Mathematical characteristics of the chitosan tablets with *Chelidonii herba* lyophilized extract (F2–F3).

Formulation	Mathematical Model								
	Zero-Order Kinetic		First-Order Kinetic		Higuchi Kinetic		Korsmeyer–Peppas Kinetic		
	*K*	*R^2^*	*K*	*R^2^*	*K*	*R^2^*	*K*	*R^2^*	*n*
F2	2.2859	0.5319	2.1004	0.2670	3.7529	0.9800	2.3284	0.7645	0.2291
F3	2.2492	0.8819	0.1419	0.4634	2.5691	0.8623	0.9104	0.9284	0.2465

*K*—dissolution constant, *R*^2^—regression coefficient, *n*—release exponent.

**Table 4:** Adhesion times of placebo (P1–P3) and chitosan tablets with *Chelidonii herba* lyophilized extract (F1–F3) to the porcine vaginal mucosa (*n* = 3; mean ± SD).

	P1	F1	P2	F2	P3	F3
Residence time (min)	<5	<5	10 ± 1	11 ± 5	240 ± 10	255 ± 20

## Supplementary Materials

The following are available online at https://www.mdpi.com/2077-0383/9/4/1208/s1, Table S1: Validation parameters.
