# Supplementary material for: Mucoadhesive Chitosan Delivery System with Chelidonii Herba Lyophilized Extract as a Promising Strategy for Vaginitis Treatment
Source: J Clin Med. 2020 Apr 22;9(4):1208. doi: 10.3390/jcm9041208 (PMC7230236; doi:10.3390/jcm9041208)
Supplement: Supplementary file 1 [file jcm-09-01208-s001.pdf]

Table 1. Validation parameters

Parameter	Results			
	Methanol		Phosphate buffer	
	Chelidonine	Sanguinarine	Chelidonine	Sanguinarine
Linearity: y = *a*x + *b*				
$a \pm S_a$	0.0122 ± 0.0003	0.7778 ± 0.0064	0.0882 ± 0.0012	0.6666 ± 0.0057
$b \pm S_b$	insignificant (α=0.05)	insignificant (α=0.05)	insignificant (α=0.05)	insignificant (α=0.05)
Correlation coefficient (*r*)	0.9997	0.9999	0.9999	0.9999
Range of linearity [μg mL^{-1}]	10.0–300.0	10.0–300.0	10.0–300.0	10.0–300.0
Intra-day precision, RSD (<5% required)				
50 [μg mL^{-1}]	0.8456	2.1580	1.5503	1.9230
100 [μg mL^{-1}]	1.2598	2.6760	0.8952	2.0518
200 [μg mL^{-1}]	0.4932	0.8348	2.4732	1.4568
Inter-day precision				
50 [μg mL^{-1}]	1.5175	2.6964	2.8871	0.7888
100 [μg mL^{-1}]	0.5162	1.4558	3.2923	2.9489
200 [μg mL^{-1}]	0.8493	0.3817	1.9178	1.8711
Limit of detection (LOD) [μg mL^{-1}]	8.99	2.68	4.58	2.79
Limit of quantification (LOQ) [μg mL^{-1}]	27.27	8.14	13.88	8.45

S_a standard deviation of the slope; S_b standard deviation of intercept, *t*, calculated values of Student's t-test, $t_{\alpha,f}$ = 2.228 critical values of Student's test for degrees of freedom f = 10 and significance level α = 0.05.
